# Review of Microfluidic Photobioreactor Technology for Metabolic Engineering and Synthetic Biology of Cyanobacteria and Microalgae

**DOI:** 10.3390/mi7100185

**Published:** 2016-10-11

**Authors:** Ya-Tang Yang, Chun Ying Wang

**Affiliations:** Department of Electrical Engineering, National Tsing Hua University, Hsinchu 30013, Taiwan; chun-ying.wang@synmikro.mpi-marburg.mpg.de

**Keywords:** cyanobacteria, photobioreactor, photosynthesis, synthetic biology, metabolic engineering, microfluidics, optofluidics, photobiology, bioreactor

## Abstract

One goal of metabolic engineering and synthetic biology for cyanobacteria and microalgae is to engineer strains that can optimally produce biofuels and commodity chemicals. However, the current workflow is slow and labor intensive with respect to assembly of genetic parts and characterization of production yields because of the slow growth rates of these organisms. Here, we review recent progress in the microfluidic photobioreactors and identify opportunities and unmet needs in metabolic engineering and synthetic biology. Because of the unprecedented experimental resolution down to the single cell level, long-term real-time monitoring capability, and high throughput with low cost, microfluidic photobioreactor technology will be an indispensible tool to speed up the development process, advance fundamental knowledge, and realize the full potential of metabolic engineering and synthetic biology for cyanobacteria and microalgae.

## 1. Introduction

**Figure 1:**
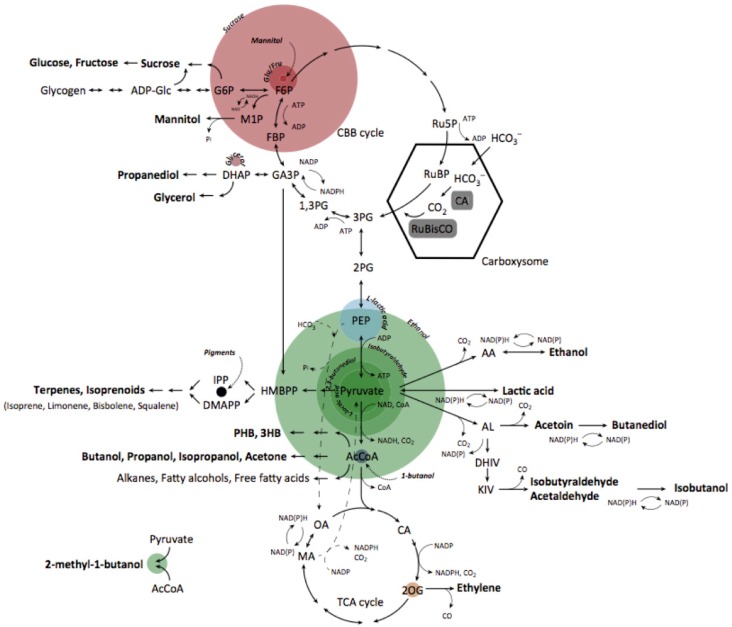
Metabolic engineering of cynaobacteria to synthesize commoidity products [3]. The carbon flux through various metabolic points is displayed. The ribulose-1,5-bisphosphate carboxylase/oxyenase enzyme (RuBisCo) catalyzes CO_2_ fixation by converting ribulose 1,5-biphosphate (RuBP) into 3PG within the carboxylsome, as indicated by the hexagon in the Calvin Bassham Benson cycle (CBB). In particular, pyruvate shows caron flux for ethanol, isobutyraldehyde, 2,3-butanediol, and lactic acid. The diameter of the circle shows the carbon flux through the respective metabolites. Reproduced with permission from Trends in Biotechnology; published by Elservier, 2015.

Recent emphasis on CO_2_ reduction and biosustainability has brought attention to photosynthetic microalgae. The microalgae, particularly cyanobacteria, are efficient organisms for producing biomass from inorganic carbon as well as important feedstocks for production of a wide range of useful compounds, including biofuels [1,2,3,4]. Cyanobacteria have the advantages of high growth rates, low nutritional requirements, and the potential for large-scale cultivation in open ponds and waters. The overall oxygenic photosynthesis process employed by cyanobacteria converts CO_2_ and water into an organic form of a carbon product with the assistance of light [5]. At the thylakoid membrane, cyanobacteria capture energy from sunlight in natural environments and generate the high-energy intermediates or cofactors, adenosine triphosphate (ATP) and nicotinamide adenine dinucleotide phosphate (NADP+), respectively. The ATP and NADP+ are utilized for the assimilation of essential nutrients in the Calvin–Benson cycle, in which the ribulose-1,5-bisphosphate carboxy/oxygenase enzyme (RuBisCo) located in carboxysome catalyzes CO_2_ fixation, as shown in Figure 1. For example, the most widely used intermediate metabolite engineers like to utilize pyruvate, the key precursor in central metabolism and product of the glycolytic pathway. “Tapping” metabolism at pyruvate allows the accumulation of large titers of ethanol [6], butanediol [7], l-lactic acid [8], d-lactic acid [9], and isobutyraldehyde [10].

Over the past decade, synthetic biology has found potential applications in diverse fields, including pharmaceuticals, biofuels, and chemical commodities An example of two successful metabolic engineering projects is the production of 1,3-propanediol (PDO) in *Escherichia coli* (*E. coli*) developed by Genencor and DuPont [11] and 1,4-butanediol (BDO) by Genomatica [12]. As time progresses, we expect similar approaches will be employed to engineer and optimize synthetic pathways for cyanobacteria. However, despite these success stories, the field of synthetic biology faces challenges in workflow automation, particularly with respect to scale up from single development projects to large, commercially viable development processes [13,14].

**Figure 2:**
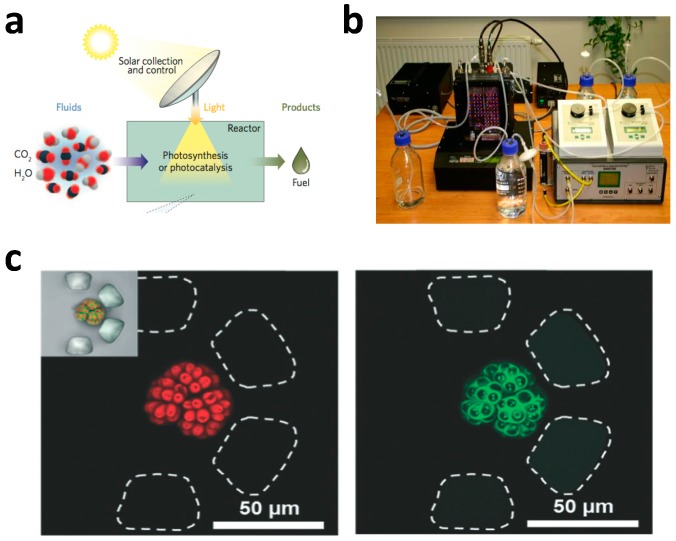
(**a**) Conceptual diagram of a photobioreactor [17]. The cyanobacteria convert CO_2_ via photosynthesis to produce useful products such as biofuel. Reproduced with permission from Nature Photonics; published by Nature Publishing Group, 2011; (**b**) Picture of a commercial photobioreactor produced by Photo System Instruments [15]. The reactor has a volume of 400 mL and a sensor for optical density measurement; (**c**) Single *Botryococcus braunii* (*B. braunii*) colony trapping in the microfluidic photobioreactor array [19]. Reproduced with permission from Lab on a chip; published by Royal Society of Chemistry, 2014. Left and right panels show the autofluorescence from cholorophyll and lipid-stained images after Nile red treatment. Reproduced with permission from Lab on a chip; published by Royal Society of Chemistry, 2012.

By definition, photobioreactors are cultivation devices for photosynthetic organisms (Figure 2a) that convert CO_2_ and light into biomass. Commercially available photobioreactors, such as Photobioreactor FMT150 from Photo System Instruments, come with different reactor volumes and allow culture growth to be monitored with integrated sensors that measure optical density and pH (Figure 2b) [15]. CO_2_ and air can also flow through the photoreactors. For example, sparging and bubbling can be used to dissolve gas into the medium. These benchtop photobioreactors have the advantages of providing rich metabolic data during the growth process, but have limited throughput. Moreover, extensive efforts are needed for sterilization, assembly, cleaning, and calibration of sensors. Advances in microfluidics cultivation technology have provided researchers in biology and biotechnology unprecedented opportunities to perform various analyses with small reagent volumes, high throughput, better spatial and temporal control over the chemical environment, and single cell resolution. The integration of microfluidics with optics has led to the field of optofluidics [16,17,18]. Erickson, Sinton, and Psaltis delineated optofluidic opportunities in sunlight-based fuel production in photobioreactors and photocatalytic systems, as well as solar energy collection systems [17]. In a typical bioprocess involving development for strain screening and evaluation, thousands of strains are screened by cultivation on plates and tubes, and a reduced number of strains are selected for scale up culture in shake flasks. Microfabricated photo bioreactors can be utilized for subsequent characterization and screening. Han et al. argued that microfabricated lab-on-a-chip systems provide both cost-effective and time-efficient opportunities for analyzing microbe-mediated bioenergy synthesis [18]. 

Here, we focused on the subsets of microfluidics that are most relevant to cultivation of algal cells; namely, flow-based microfluidic large-scale integration (mLSI), droplet microfluidics, and digital microfluidics (DMF) based on electrowetting. mLSI is largely based on polydimethylsiloxane (PDMS) material with multilayer soft lithography [20] and offers the researchers to design the fluidic circuits of almost arbitrarily complexity. In multilayer soft lithography, one layer typically serves as the flow layer and another as the control layer, with channels pressurized by an external pressure source. The integration of the control layer and flow layer with valves forms the building block of mLSI [20]. Droplet-based microfluidics involves the generation and manipulation of discrete droplets at high throughput [21]. This method produces droplets in the picoliter to microliter diameter range, which can be transported, merged, and analyzed. Each droplet serves as a reaction vessel with high surface-to-volume ratio. Unlike mLSI, droplet microfluidics has the capacity to perform a large number of reactions in a repetitive manner and train format without increasing chip complexity. Parallel processing and experimentation can easily be achieved to allow the acquisition of large amounts of data because a large number of droplets can be formed. The term digital microfluidics is used to describe the control of droplet position based on electrowetting [22,23]. In electrowetting, a fluid is placed on an electrode coated with an insulator that has a surface treated to be hydrophobic. A potential is applied across the insulator to make it charged and therefore attractive for the fluid to wet the surface. Currently, digital microfluidics has been used for a wide range of laboratory analyses. Similar to mLSI, DMF enjoys the same benefits of low reagent volume (in the picoliter to microliter range) and high capacity for parallelization and automation. Moreover, it can be easily integrated with other analytic techniques. However, the droplets in DMF devices are often exposed to ambient conditions; therefore, the evaporation of reagents is a problem, especially during long-term cultivation of cells.

## 2. Review of Main Body of Research

### 2.1. General Comments on Microfluidic Photobioreactors

Here, we provide a review of the recent progress in opportunities afforded by microfluidics for cyanobacteria and microalgae from the perspective of metabolic engineering and synthetic biology. This paper primarily focuses on microfluidic cultivation technology because this is most relevant for investigation of the physiology and metabolism of cyanobacteria. Key factors for design of microfluidic photobioreactors includes the light illumination, CO_2_ gas delivery, nutrient medium supply, and mechanical form factors of the reactor. The parameters for light illumination include the intensity, wavelength, and temporal and spatial patterns. In addition, monitoring the growth of the cells during the cultivation and end point detection of the desired product are also crucial. As the exact device design and experimental protocol depends on the model organisms used, one need to consult the existing literature [4]. We also review the progress in microfluidic photobioreactor technology for metabolic engineering and the synthetic biology of cyanobacteria and microalage. Before reviewing the microfluidic photobioreactor, we noticed that the majority of the work on microfluidic cultivation has involved non-photosynthetic bacteria. Extensive efforts have been made to design microfluidic bioreactors for non-photosynthetic microbial cultivation [24] and cell culture [25] in the form of microchemostats [26,27,28], serial dilution bioreactors [29], flow-based chemostats [30,31,32], biofilm flow reactors [33,34,35], and droplet reactors [36]. Here, we review microfluidic photobioreactors and separate them according to different platform technology.

### 2.2. Microfluidic Photobioreactor Based on Microplate and Agar 

**Figure 3:**
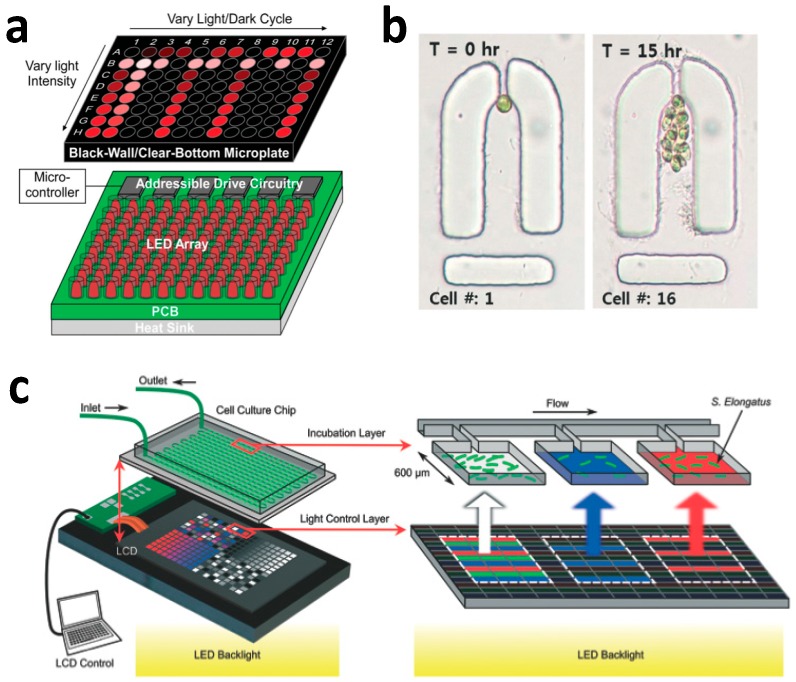
High throughput screening microfluidic platform (**a**) 96-well microplates [37]. An array of LEDs is utilized in conjunction with a microcontroller to provide light illumination; Reproduced with permission from Lab on a chip; published by Royal Society of Chemistry, 2012; (**b**) Single-cell capture sites [41]. Single-cell resolution growth profile of *C. reinhardtii* showing cell division inside the cell trap site for a 15-h period; Reproduced with permission from Lab on a chip; published by Royal Society of Chemistry, 2015; (**c**) Liquid crystal display (LCD) pixel-based photobioreactors [44]. The platform consists of a PDMS-on-glass cell culture chip, a programmable LCD screen and an LED array backlight to deliver the multiplexed illumination to cyanobacteria. The irradiance intensity, time variance and spectral composition can be individually controlled for each experiment. Reproduced with permission from Lab on a chip; published by Royal Society of Chemistry, 2015.

Chen et al. used a 96-well microplate integrated with a LED light source for high-throughput studies of light-dependent growth rates and characterized photosynthetic efficiency in the model organism Dunaliella tertiolecta, a lipid-producing algae as shown in Figure 3a [37]. They claimed to reduce the screening time from two years using conventional tools to less than two weeks by conducting 96 photoirradiance experiments in parallel. However, it is reported that growth of cyanobacteria in 96 well is poor as compared to 6 well plates or 24 well plates [4]. Teng et al. elucidated the mechanism for robust circadian oscillations in growing *Synechococcus elongatus* (*S. elongates*) [38]. To accomplish this, they used a single-cell chemostat based on an agarose pad [31,39] patterned with submicron grooves to monitor the oscillation of a wild-type strain and identify the role played by a transcription translation regulation (TTR) circuit in enhancement of stability of circadian clocks.

### 2.3. Microfluidic Photobioreactor Utilizing Flow-Based mLSI Technology

We next described a photobioreactor based on PDMS mLSI technology. PDMS has the advantage of gas permeability; therefore, CO_2_ can diffuse into the culture chamber. The PDMS material can also prevent the evaporation of the medium for an extensitve time period for cultivation of cells if the chip is kept in a humidified environment. Holcomb et al. demonstrated the biocompatibility of PDMS devices with microalgae by culturing microalgae and used fluorescent dye for lipid staining [40]. With a power-free valve, the device can support culture of *Tetraselmis chuii* (*T. chuii*) for up to 3 weeks. Han et al. demonstrated a high-throughput microfluidic microalgal photobioreactor array capable of applying 64 different light conditions for *Botryococcus braunii* [19]. The device is composed of four poly(dimethylsiloxane) (PDMS) layers stacked on top of each other, a microalgae culture layer, a light intensity control layer, a light–dark cycle control layer, and a light-blocking layer. By co-flowing deionized (DI) water and black dye through the light intensity layer, the gradient generator produces eight different concentrations of black dye and hence eight different light intensity conditions. Similarly, the control of light–dark cycles is based on selectively filling each microfluidic channel in the light–dark cycle control layer with either DI water or black dye. They measured the growth and oil production of *B. braunii* for 12 days under 16 different light exposure conditions. Subsequently, the same group refined the device design and reported a high throughput microfluidic single-cell screening platform as shown in Figure 3b [41]. The developed platform consists of 1024 single-cell trapping units for the unicellular microalga *Chlamydomonas reinhardtii* (*C. reinhardtii*) and measured the growth rates using chlorophyll as an authofluorescence marker and intracellular lipid accumulation by staining the cell with Nile red fluorescenct dye. The doubling time of *C. reinhardtii* was determined to be 6–8 h, which was consistent with conventional bulk measurement results. 

**Figure 4:**
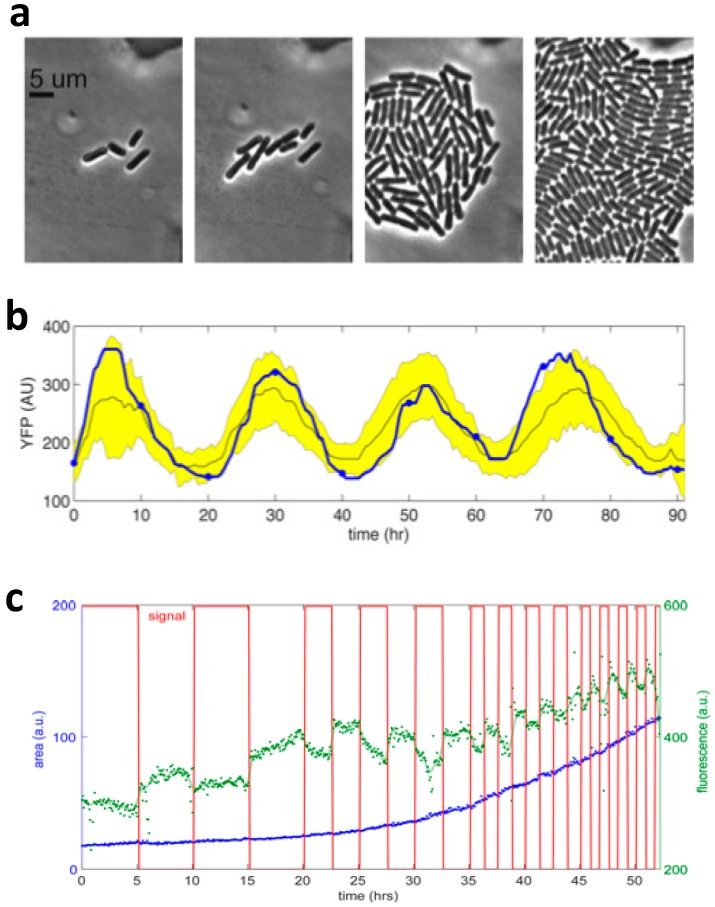
Microfluidic platform for long term monitoring of algae in a dynamic environment [43]. (**a**) Time-lapse image of *S. elongates* in a microfluidic chamber to show growth; (**b**) The fluorescence level of YFP of 134 cells from single cell tracking of circadian rhythms of gene expression of *S. elongates.* The blue line and yellow shaded region are the mean and the standard deviation, respectively; (**c**) Chlorophyll fluorescence of *Chlorella sorokiniana* (*C. sorokiniana*) under dynamic simulation with pulses of ammonia. Chlorophyll fluorescence (green line) decreased when ammonia was introduced with medium void of nitrogen. The total cell area is indicated by the blue line. Reproduced with permission from ACS Synthetic Biology; published by American Society of Chemistry, 2016.

A very similar perfusion platform was also developed by Eu et al. to cultivate motile microalgal cells. The platform consists of a 2 × 4 array of perfusion chambers with 2-μm-tall pillar structures to prevent cells from escaping [42]. The chemical environment in each chamber was independently controlled and used to monitor lipid production under nitrogen depletion, phototaxis behavior in the absence of calcium ion and cytotoxic effects due to herbicides. Luke et al. developed a Dial-a-Wave (DAW) microfluidic platform for long term monitoring of cyanobacteria and microalgae [43]. In their system, cells are confined in a microfluidic chamber with height slightly lower than the dimensions of the cell and allowed to grow under the perfusion of nutrient medium. Such monolayer confinement avoids the shading effect typically encountered in macroscopic photobioreactors and ensures the efficient illumination of cells. The reported doubling times in microfluidic devices are similar or shorter than those in bulk culture under the same light intensity. For example, *S. elongates* PCC 7942 can grow via phototrophic metabolism, although the phototropic growth rate is relatively lower, ~0.12 h^−1^, with a corresponding doubling time of ~6 h. Their devices also track circadian rhythms in *S. elongatus* using yellow fluorescence protein (YFP) as the reporter from gene expression under the control of kaiBC promoter, as shown in Figure 4b. Dynamic stimulation is also demonstrated by pulsing of 100 ppm ammonia at different periods and observing the chlorophyll authofluorescence, as shown in Figure 4c.

### 2.4. Microfluidic Photobioreactor Based on Droplet and Digital Microfluidics

The microfluidic photobioreactor based on droplet format in PDMS materials has also been developed for very high throughput screening experiments. For example, microfluidics is ideally suited for single cell electroporation, because it can be used to overcome several inherent drawbacks of bulk electroporation. First of all, only a relatively low potential is needed to generate a high electric field strength with microelectrodes and therefore minimized the joule heating. Secondly, heat dissipation is fast in microfluidic channel owing to the large surface area-to-volume ratio and as a result increase the cell viability. Both effects can minimize the temperature increase during the electroporation and increase cell viability. Qu et al. developed a PDMS-glass to perform electroporation for *C. reinhardtii* [45]. The device consists of a flow focusing microstructure to generate cell-encapsulating droplets and a serpentine channel to enhance fluidic mixing. The transformation efficiency was shown to be more than two orders of magnitude higher for the wild-type cell than bulk phase electroporation. The maximum transmembrane potential for on-chip electroporation is about ~295 mV and for comparison, 0.75 kV is used for a commercial electroporator (Bio-Rad). 

Abalde-Cela et al. have developed a screening platform based on droplets for ethanol producing cyanobacteria that utilizes a customized enzyme detection assay and fluorescence [46]. The technique is based on an enzyme assay that converts ethanol into a highly fluorescent compound. The growth kinetics for *Synechocystis* sp. PCC6803 were measured in microdroplets. A fully integrated chip based on droplet microfluidics was also developed by Han et al. [47]. The device consists of a culturing region, an on-chip staining region, and a rinsing and analysis region. After cell cultivation, the cells encapsulated in the microdroplets are synchronized and merged with Nile red droplets to stain the lipids. Finally, the droplets pass through a rinsing region for oil quantification. Hammar et al. have developed a droplet microfluidic workflow for single cell analysis and sorting of l-lactate-producing strains of *Synechocystis* sp. PCC 6803 [48]. A UV mutagenized population was sorted using fluorescent-activated droplet sorting and the separation of low- and high-producing strains is demonstrated. More importantly, the experimental data with single cell resolution revealed population heterogenity in photosynthetic growth and lactate production as well as the metabolically stalled cells. 

Now, we discuss the microfluidic photobioreactors based on DMF. Au, Shih and Wheeler have developed a DMF platform as a micro bioreactor and reported 5-day culture of algae [49]. This platform has been future optimized with several design features to allow fully automated, multiplexed analysis with significant reductions in pipette steps [50]. The device features an active reservoir structure to maintain homogeneous cell density and a customized device layout for controlling a wide range of various droplet sizes. The readout is conducted in the detection zone using a standard multiwell plate reader to allow parallel optical measurement. For confirmation of its functionality, the device is used to identify the optimal illumination conditions for biofuel production from *Cyclotella cryptica*.

### 2.5. Microfluidic Photobioreactor with Alternative Illumination Method

**Figure 5:**
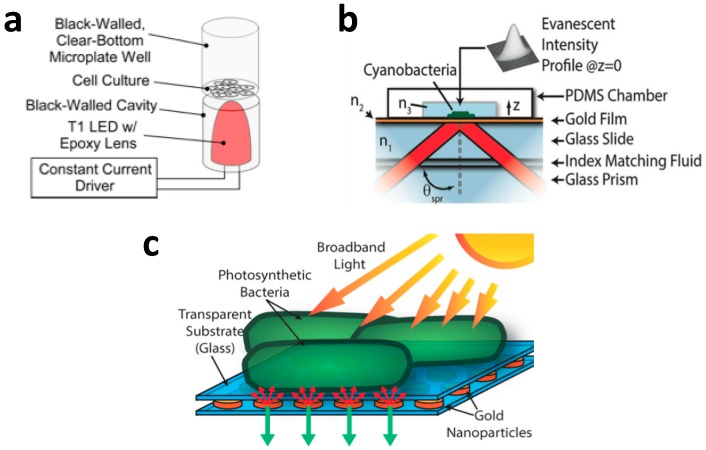
Light coupling mechanisms. (**a**) Direct illumination from a LED [37]. Reproduced with permission from Lab on a chip; published by Royal Society of Chemistry, 2012; (**b**) Surface plasmon illumination [55]. Cyanobacteria are placed on a gold film and light is coupled via a glass prism in the Kretschmann configuration; Reproduced with permission from Applied Physics Letter; published by American Institute of Physics, 2012; (**c**) Localized surface plasmon resonance (LSP) coupling [56]. Cyanobacteria are placed on an array of gold nanoparticles, which are designed to optically resonate near the absorption maxima of pigments to reflect useful light to these microorganisms. Reproduced with permission from Applied Physics Letter; published by American Institute of Physics, 2014.

One research direction is to explore alternative ways to illuminate cyanobacteria. In this regard, researchers have resorted to ideas from micro and nano optics (Figure 5). Erickson et al. demonstrated slab waveguide photobioreactors for *S. elongates* PCC 7942 [51,52]. These reactors use an evanescent wave to improve the illumination uniformity for cyanobacteria. They coupled a laser light with a wavelength of 660 nm to the slab waveguide, which is actually the coverslip substrate of thickness 150 µm in the PDMS microfluidic chip. They then characterized the growth rate by measuring the optical density at 750 nm off chip and concluded that there was a 12-fold improvement in volume productivity. One possibility is to use the surface plasmon resonance [53,54]. Surface plasmon is the collective oscillation of electrons typically in metallic materials and properly engineered plasmonic nanostructures can effectively confine the optical field well below the optical wavelength using localized optical modes. The local surface plasmon fields are also enhanced by a factor of *Q* when under external excitation (*Q* is defined in terms of the real and imaginary parts of the metal’s permittivity (ɛ_m_) as *Q* = −*Re*·ɛ_m_/*Im*·ɛ_m_). For noble metals, the maximal value of *Q* ranges from 10 up to 100. Sinton et al. showed that the surface plasmon resonance evanescent field can be used to enhance the growth of *S. elongatus* biofilm [55,56]. They coupled laser light at a wavelength of 633 nm via a glass prism in Kretschmann configuration to grow a thick biofilm. The high-intensity evanescent field penetrated less than 1 µm into the media, but led to an improvement in the volume density of the cyanobacteria cells. In a similar study, Sinton et al. demonstrated that excitation of photosynthetic *Synechococcus bacillaris* biofilms can provide electricity directly [57]. The biofilm was attached with an electrode and placed on a gold film for plasmonic excitation via the Kretschmann configuration at λ = 670 nm. Sinton et al. demonstrated a microfluidic photobioreactor with LED pixels as the illumination source [44]. The platform consists of a PDMS culture chip, a programmable LCD screen and an LED array backlight to individually control the irradiance intensity, time variance and spectral composition of each individual chamber.

## 3. Future Perspectives

### 3.1. Integration with Current Workflow of Synthetic Biology

Genome engineering of cyanobacteria is critical to metabolic engineering and synthetic biology [58]. Currently, the development of genome engineering tools for cyanobacteria lags far behind that of other model organisms such as *E. coli*. Standardized components such as promotors require extensive characterization to produce predictable results. Current automation for gene assembly relies on robotic technology, which is prohibitively expensive [58,59,60]. For example, the consumable cost and hands on time for DNA assembly and cloning using traditional liquid-handling robotic automation is still prohibitively expensive, and significant capital investment is required. In contrast, modest infrastructure and inexpensive microfluidic devices are suitable platforms for widespread use. Lin et al. reported the use of digital microfluidics for DNA ligation with single DNA fragment insertion [60]. Linshiz et al. used valve-based channels to carry out Golden gate and Gibson assembly with insertion of up to eight DNA fragments [61]. More recently, Shih et al. reported a versatile microfluidic device to conduct DNA assembly based on three commonly used DNA assembly protocols that combined digital and droplet microfluidics [62]. The DNA assembly region consists of digital microfluidic devices with 76 electrodes, while the incubation and queuing takes place in a serpentine channel. Electroporation is used to transform the microbes using electrodes. In particular, the field intensity is varied in the range of 1000−2000 V/cm to optimize the intensity that will result in the highest transformation efficiency for our microfluidic setup. For *Escherichia coli* a maximum efficiency 4.58 × 10^3^ cfu/ng is achieved at 1800 V/cm. For yeast, lower fields (~1200 V/cm) yielded a maximum efficiency of 1.90 × 10^3^ cfu/ng of DNA.

### 3.2. Synthetic Photobiology and Optogenetics

Optical means to control genetic circuits provide a more precise method than chemical effectors, which are the current standard, for controlling gene circuits in synthetic biology [63]. Parameters such as wavelength and intensity can be precisely tuned to achieve the desired control over the gene expression. Light sensing microorganisms can be engineered by taking existing sensors from a light-sensing organism such as cyanobacteria or plants and fusing them into two component systems in the host microorganisms. In natural environments, cyanobacteria possess sensors used for chromatic adaption to optimize their light utilization, and such sensors can be used for light sensing. Voigt et al. created a light sensor that functions in *E. coli* by engineering a chimaera protein that uses a phytochrome from a cyanobacterium [64,65]. Tabor et al. subsequently created and optimized a few light sensing *E. coli* using green/red light and red/infrared light [66]. Ohlendorf et al. also created a one-plasmid system using blue light to regulate gene expression [67,68]. Although there has been significant progress in developing and optimizing such optically addressable genetic circuits, the current standard protocol is still too cumbersome and labor intensive. For example, Tabor’s group created a light tube array to conduct measurements using a procotol that involves microbial cultivation under the illumination of light, freezing the metabolic activity at low temperature and antibiotic and fluorescent measurement in a flow cytomoter [69]. The aforementioned microfluidic photobioreactors can serve as ideal platforms to characterize such light-sensing microorganisms in automated fashion and with single cell resolution.

### 3.3. End Point Titration and Metabolic Flux Measurement

In the current standard process flow, the end product titration relies on HPLC (high-performance liquid chromatography), which requires significant amounts of sample (~100 µL). The results are also limited to end point detection. Real-time profiling of metabolic activity has been demonstrated with direct injection of living bacteria into a high-resolution mass spectrometer [70], but such a capability is only available in a very limited fashion. The metabolic flux of cyanobacteria has also been measured using isotope-labeling techniques [71]. Moreover, the absolute concentration of metabolites has been measured to obtain a global understanding of the metabolome for *E. coli* [72]. These recent developments are very exciting, but they all employ techniques that are cumbersome and require expensive equipment. Accordingly, it is desirable to have low cost, in situ monitoring of product production integrated with microfluidics. For example, a single-cell Raman spectra-based approach is rapid, label-free, non-invasive, low-cost, and potentially able to simultaneously track multiple metabolites in individual live cells; therefore, such a method should enable many new applications. This method also bypasses the slow, cumbersome culture step of microalgae. Zhang et al. described a method for direct, quantitative, in vivo lipid profiling of microalgae using single-cell laser-trapping Raman spectroscopy for several oil-producing algal species of interest, including *B. braunii*, *Neochloris oleoabundans*, and *C. reinhardtii* [73,74]. This technique afforded in vivo quantitative spectroscopy from single living cells without any preparation step and is much more convenient than conventional Raman measurement technique performed on bulky, dried, or immobilized algal samples. Huang et al. used the starch-producing unicellular microalga *C. reinhardtii* as a model and employed a customized Raman spectrometer to capture the Raman spectra of individual single cells [74]. 

## 4. Conclusions

In conclusion, microfluidics can bring a great deal to the field of metabolic engineering and synthetic biology of cyanobacteria. The main driving force will be to develop a platform for identifying highly valued strains with rapid turnaround times. First of all, as the light is necessary ingredient for cyanobacteria and microalgae, optimization of light illumination for photosynthesis have been explored extensively and form a line of research direction on its own [51,52,53,54,55]. Unlike the macroscopic photobioreactors, for which self shading is almost avoidable, the microfluidic photobioreactor can be designed with uniform light intensity across the cultivation volume. Although advances have been made to make microfluidic photobioreactors in several microfluidic technologies, this field is still in its fledging phase. So far, each microfluidic technology such as mLSI, droplet microfluidics, DMF has their own unique advantages as well as their shortcomings. For example, if the throughput for screening is the major concern, the droplet microfluidics is the winner [46,47]. However, the cells are cultivated within the droplet with a fixed amount of nutrient medium and will not be in a well-defined constant chemical environment as the metabolic waste accumulated. The flow based reactors based mLSI technolgy with PDMS material provide well-defined chemical environments and with single cell resolution is still the most ideal choice to obtain the characterization of genetic circuit [43]. DMF seems to be a unnatural choice for cultivation of cyanobacteria and microalgae mainly because prevention of the evaporation of medium require extensive engineering [50]. However, it will be most likely be winner solution for gene assembly [60,62]. Still, current research efforts in microfluidics are focused on demonstrating proof of concept devices and experiments. The results reported in the literature remain isolated from the actual workflow of strain improvement through synthetic biology approaches. Filling this gap will be important to advancing microfluidic technology. For example, a microfluidic device capable of testing the cell culture condition under a large number of conditions with different CO_2_ gas and medium composition will be very desirable and has been lacking although there are reports using Dertinger mixing devices to create gradient [75]. In Table 1, we summarize the challenges in the current synthetic biology workflow and potential solutions provided by microfluidic technology. The current cumbersome workflow to insert and verify a synthetic pathway or a genetic circuit via gene assembly technique into the cyanbacteria presents tremendous opportunities for microfluidic technology. One fundamental bottleneck in turnaround time is the long cultivation time for cyanobacteria. This can be overcome by either bypassing the cultivation step using sensitive spectroscopy tools or monitoring the cyanobacteria at the single cell level. On one hand, it seems that this field has a clear roadmap to follow and there is no need to reinvent the wheel, as many microfluidic device designs are well characterized and readily available for specific experimental needs. On the other hand, the microfluidic technology can offer unprecedented experimental resolution to advance our fundamental understanding of genetic circuits of cyanobacteria [37,43], which will subsequently help realize the full potential of syntheic biology and metabolic engineering of cyanobacteria. For example, the effect of molecular fluctuation at the single enzyme level on growth has been demonstrated for *E. coli* using microfluidic devices [76]. It would be interesting to see if the same effect could be measured for individual cyanobacteria. Another example is to utilize the microfluidic technology to understand the cellular heterogeneity. It is known genetically identical cell can exhibit phenotypic heterogeneity and such effects can be monitored with microfludic devices with single cell resolution and high throughput [47,77]. Another example is the development of a high-efficiency synthetic carbon fixation pathway to replace the Calvin–Benson cycle based on the RuBisCo enzyme. Milo et al. investigated the possibility of a synthetic carbon fixation pathway, and experimental processes along this line are ongoing [78,79]. Microfluidic devices can offer high throughput platforms to test cyanobacteria under a variety of growth conditions. Although there is still a long way to go, we believe that the results of these efforts will be very rewarding. Genetically modified microorganisms hold great promise from a biotechnological point of view, and may become a green production technology for biofuel and commodity chemical production under controlled conditions.

## Figures and Tables

**Table 1:** Key microfluidic technologies for investigation of challenges present in the metabolic engineering and synthetic biology workflow.

Work Flow	Challenges	Microfluidic Technology	References
Gene assembly	Fast, accurate, construction of large genetic devices	Microfluidics DNA synthesis and assembly with integrated bacterial transformation	[60,62]
Verification	Large scale screening Dynamic chemical environment	Microfluidic photobioreactor supporting long term cell growth and single cell monitoring	[41,42,43]
End production titration	Low cost, in situ measurement to replace current technology	1. Enzyme assay with fluorescent detection 2. Spectroscopy such as Raman integrated with microfluidics	[46,48,73,74]
